# White Lupin Drought Tolerance: Genetic Variation, Trait Genetic Architecture, and Genome-Enabled Prediction

**DOI:** 10.3390/ijms24032351

**Published:** 2023-01-25

**Authors:** Luciano Pecetti, Paolo Annicchiarico, Margherita Crosta, Tommaso Notario, Barbara Ferrari, Nelson Nazzicari

**Affiliations:** Council for Agricultural Research and Economics (CREA), Research Centre for Animal Production and Aquaculture, Viale Piacenza 29, 26900 Lodi, Italy

**Keywords:** drought stress, genotype × environment interaction, genomic selection, GWAS, grain yield, phenology, plant adaptation

## Abstract

White lupin is a high-protein crop requiring drought tolerance improvement. This study focused on a genetically-broad population of 138 lines to investigate the phenotypic variation and genotype × environment interaction (GEI) for grain yield and other traits across drought-prone and moisture-favourable managed environments, the trait genetic architecture and relevant genomic regions by a GWAS using 9828 mapped SNP markers, and the predictive ability of genomic selection (GS) models. Water treatments across two late cropping months implied max. available soil water content of 60–80% for favourable conditions and from wilting point to 15% for severe drought. Line yield responses across environments featured a genetic correlation of 0.84. Relatively better line yield under drought was associated with an increased harvest index. Two significant QTLs emerged for yield in each condition that differed across conditions. Line yield under stress displayed an inverse linear relationship with the onset of flowering, confirmed genomically by a common major QTL. An adjusted grain yield computed as deviation from phenology-predicted yield acted as an indicator of intrinsic drought tolerance. On the whole, the yield in both conditions and the adjusted yield were polygenic, heritable, and exploitable by GS with a high predictive ability (0.62–0.78). Our results can support selection for climatically different drought-prone regions.

## 1. Introduction

White lupin (*Lupinus albus* L.) is a Mediterranean grain legume that used to be a major crop in various regions of the Roman Empire because of its ability to adapt to and improve infertile soils [1]. A recent surge of interest in its cultivation as a rain-fed food or feed crop in Europe is justified by its protein content that is close to 40% and other favourable quality traits of its seed [2,3,4], which can respond to the increasing demand for healthy and nutritious plant-based food [5] and high-protein feedstuff [6]. In particular, the exploitation of white lupin seed as a component of functional, healthy, or vegan food is favoured by its good content of essential amino acids and several useful techno-functional properties [7], the positive effects on human health that it can exert with respect to diabetes and glycaemia, hypertension, cardiovascular diseases, and obesity [8], and 8–12% content of oil with excellent nutritional characteristics [9]. The potential value of white lupin for feed protein production was confirmed by its greater crude protein yield per unit area compared with other cool-season grain legumes, such as pea (*Pisum sativum* L.), faba bean (*Vicia faba* L.), and narrow-leafed lupin (*Lupinus angustifolius* L.), across climatically-contrasting, autumn-sown environments of southern Europe [10]. However, insufficient yielding ability limits the spread of this crop crucially [11].

White lupin adaptation to severe drought has paramount importance in Mediterranean-climate areas, where this stress occurs in coincidence with critical reproductive stages. Drought stress is predicted to increase throughout the Mediterranean basin and to expand northward and eastward into Europe, owing to lower rainfall and rising evapotranspiration caused by climate change [12]. The available information on the extent of white lupin genetic variation for drought tolerance is modest and concerns mainly landrace material. A fairly narrow variation was observed in germplasm from Portugal [13]. Variation for this trait was reported in Egyptian germplasm [14], while a few accessions of Italian origins were found to be more drought tolerant than local germplasm in Egypt [15]. Annicchiarico et al. [16] reported large yield variation among accessions of a world landrace collection grown in a moderately favourable Mediterranean environment, along with the definite superiority of several landrace accessions over a set of control varieties. Variation for specific adaptation to severe drought, as indicated by a fairly large genotype × environment interaction (GEI) for grain yield across drought-prone and moisture-favourable managed environments, was reported by Annicchiarico et al. [17] for a set of landraces having different geographic origins and similar phenology. Moderate GEI across these environments emerged also for individual genotypes sorted out of landrace accessions [18]. Finally, large GEI was reported for a small set of breeding lines across subcontinental-climate and Mediterranean-climate sites of Italy [19].

The tolerance of cool-season grain legumes to the terminal drought that features Mediterranean-climate regions is typically associated with an early phenology, but the exploitation of this drought stress-escape mechanism in autumn-sown environments may be limited by the lower winter hardiness of early-flowering germplasm [20,21]. When targeting such environments, it may be useful to assess and exploit the genetic variation for intrinsic drought tolerance obtained by partialling out the effect of phenology on genotype yield responses [22]. As an alternative, more demanding avenue, one may dissect and exploit the variation for single traits that contribute to drought avoidance or drought resistance [23,24].

The scope for molecular marker-based selection has widened considerably after the development of next-generation sequencing techniques, such as genotyping-by-sequencing (GBS; [25]), which allows genotyping large germplasm sets by thousands of single nucleotide polymorphism (SNP) markers at a relatively low cost. Książkiewicz et al. [26] confirmed the ability of GBS to generate thousands of polymorphic SNP markers for white lupin genetic analyses and highlighted its value for a genome-wide association study (GWAS). Identifying and exploiting putative QTL (quantitative trait loci) hotspots of the genome that are associated with drought tolerance led to enhanced crop yield under drought in two other cool-season grain legumes, such as pea [27] and chickpea [28]. In the presence of a definitely polygenic trait genetic architecture revealed by a GWAS, which is likely to emerge for complex traits such as crop yield or drought tolerance, an alternative molecular breeding strategy is represented by genomic selection (GS). This strategy combines phenotyping and genotyping data of a genotype sample (training population) representing a target genetic base (reference population) into a statistical model for the prediction of breeding values in future plant selection [29,30]. Pioneer examples of GS for grain legume drought tolerance improvement were provided for pea [22] and chickpea [24], and a proof-of-concept study confirmed the ability of GS to identify drought-tolerant pea lines [27]. Encouraging results for GS of white lupin yield emerged in Annicchiarico et al. [18], who reported predictive ability values (as Pearson’s correlation between predicted and observed values based on intra-environment cross-validations) in the range of 0.47–0.76 for geographically-diversified landrace material evaluated in climatically-contrasting regions or across moisture-favourable and severely drought-prone managed environments. A second study on landrace germplasm reported high to moderate predictive ability (0.49–0.85) for a set of morphophysiological traits [31].

White lupin studies on drought tolerance variation, GEI and genome-enabled predictive ability are scanty. In addition, their results relative to landrace germplasm are not necessarily meaningful to breeding programs, which select inbred lines whose variation for yielding ability and drought tolerance on the one hand and for molecular markers on the other may be smaller than those of geographically-diversified landrace material. This study focused on a reference population of inbred lines generated by crossing each of four elite sweet-seed cultivars or breeding lines with each of four elite landrace accessions of different geographical origins. Its main objectives were (a) to assess the phenotypic variation for adaptation to severe drought and the extent of GEI for grain yield, aerial biomass, harvest index, and the onset of flowering across drought-prone and moisture-favourable managed conditions, (b) to investigate the genetic architecture and the presence of highly relevant genomic regions for grain yield under severe drought and favourable conditions and intrinsic drought tolerance by a GWAS, and (c) to assess the ability of different GS models to predict genotype yield responses, drought tolerance, and the onset of flowering.

## 2. Results

### 2.1. Adaptive Responses

The reduction of water supply in the drought-prone managed environment relative to the moisture-favourable one, which amounted to 53% over the crop cycle (170 vs. 360 mm) and 79% over the two-month period of stress application (50 vs. 240 mm), determined average reductions of 61% for grain yield (0.88 vs. 2.28 t/ha) and 66% for aerial biomass (grain plus straw: 3.13 vs. 9.13 t/ha). The somewhat lower penalty suffered by grain yield relative to aerial biomass was associated with a concurrent, slight increase of the harvest index under stress (0.281 vs. 0.250) (Table 1). Drought stress also caused an anticipation of 2.8 days of the mean onset of flowering (Table 1).

The genetic variation among inbred lines was significant for all traits in each environment (*p* < 0.01) but tended to decrease under drought relative to moisture-favourable conditions for grain and straw biomass according to the genetic coefficient of variation values (Table 1). The broad-sense heritability, however, was comparable across conditions, except for the lower value under favourable conditions of the harvest index (Table 1). Line grain yield values ranged from substantial crop failure (0.09 t/ha) to 1.64 t/ha under stress, and from 0.45 to 4.68 t/ha under favourable conditions. The line drought susceptibility index (DSI) ranged from 0.18 to 1.74.

**Figure 1:**
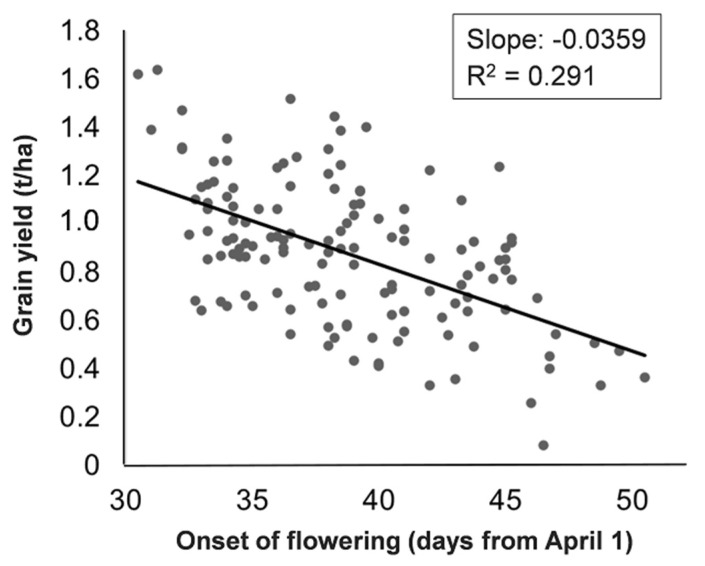
Linear regression of grain yield under drought stress as a function of onset of flowering for 138 white lupin inbred lines.

Line grain yield under stress exhibited an inverse linear relationship with the onset of flowering (*p* < 0.01), across about 20 days of line phenology range (Figure 1). The regression *R*^2^ value suggested that drought stress escape accounted for nearly 30% of the line yield variation under stress, with an average yield loss of 0.036 t/ha per day of delayed flowering (Figure 1). The adjusted grain yield computed as the yield deviation from regression, which acted as an indicator of intrinsic drought tolerance, varied from −0.52 to 0.57 t/ha (Figure 1). This measure displayed broad-sense heritability of practical interest for breeders, although its value was somewhat lower than that for grain yield under stress (0.59 vs. 0.70; Table 1).

A combined analysis of variance (ANOVA) indicated the occurrence of GEI across managed environments (*p* < 0.01) for all traits except the onset of flowering. However, a significant lack of genetic correlation (*r_g_*) for line responses across environments (as indicated by the significant deviation from the unity value of *r_g_* at *p* < 0.05) was only found in grain yield and harvest index (Table 2), suggesting that GEI for the other traits was essentially due to the heterogeneity of genotype variance across environments. The unity value of *r_g_* for the onset of flowering indicated a perfect consistency of line phenology differences across environments (Table 2). The GEI effects for grain yield and harvest index were correlated (*r* = 0.43, *p* < 0.01), indicating that genotypes that displayed relatively better performance under drought tended to increase their harvest index under drought more than drought-susceptible material.

Line grain yield was positively correlated with straw biomass and harvest index, and negatively correlated with the onset of flowering, under both drought stress and favourable conditions (*p* < 0.01; Table 3). However, the relationships of grain yield with harvest index and phenology were closer under drought stress than under favourable conditions (Table 3), in agreement with the importance of earlier phenology for stress escape and the exploitation of GEI for greater harvest index under drought by drought-tolerant lines that were suggested by previous results. 

The DSI exhibited just a slightly negative relationship with line yield under stress (*r* = −0.14, *p* < 0.10) while being closely associated with line yield in favourable conditions (*r* = 0.62), indicating that its values depended mainly on the line-yielding ability under favourable conditions and would, therefore, be of limited value for selection of drought-tolerant germplasm (unlike adjusted grain yield values, which displayed high correlation with grain yield under stress: Table 3). As a consequence, DSI was neglected by following genome-focused analyses. 

On average, the progeny lines of the landrace La646 displayed the highest intrinsic drought tolerance according to adjusted yield data (Table 4). This feature, which was associated with late flowering, led to mean grain yield under drought of its progeny lines that was comparable with that of the progeny lines of the Moroccan line L27PS3, of which the good yielding ability under stress was due to very early flowering rather than intrinsic drought tolerance (Table 4). High mean grain yield under drought due to early flowering was also shown by progenies of the landrace LAP123. In contrast, poor mean yield in this condition associated with high susceptibility to drought tolerance was exhibited by progenies of the landrace Gr56 and, to a lesser extent, those of line MB-38 (Table 4). 

### 2.2. Trait Genetic Architecture

Genotyping-by-sequencing (GBS) of the DNA samples generated, on average, 1.93 million reads per sample. After alignment, SNP calling, and quality filtering, we obtained a set of 19,024 markers, which were further filtered for missing rates. We retained for linkage disequilibrium (LD), population structure, and GWAS analyses 9828 polymorphic SNP markers that mapped on the *Lupinus albus* genome released by Hufnagel et al. [33], after imposing the thresholds of 30% maximum missing rate per marker and 50% maximum missing rate per sample. For GS analyses, we also envisaged other thresholds of missing rate per marker (i.e., 15% and 20%).

On average, the LD reached half of its 90th percentile (*r*^2^ = 0.38) at 811 bp, with values for the single chromosomes that ranged from 338 bp for the chromosome 12 to 1592 bp for the chromosome 10 (Supplementary Figure S1).

**Figure 2:**
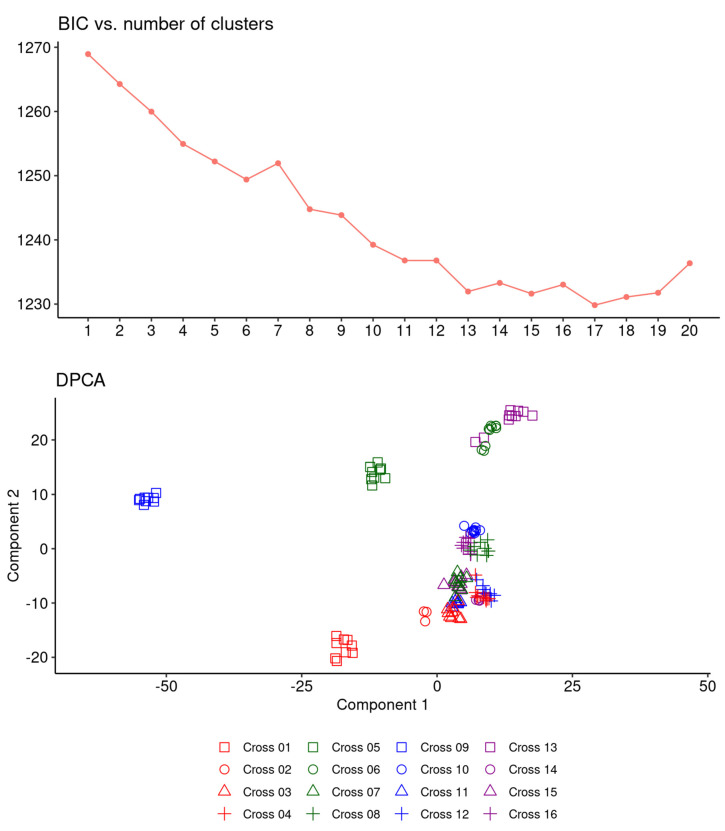
Assessment of population structure by a discriminant principal components analysis (DPCA). The top panel shows the Bayesian Information Content (BIC) as measured after clustering the samples by the k-means algorithm for increasing levels of K. The bottom panel shows the first two DPCA components for the selected K level. The symbol shapes represent the sweet-seed parent line (Lucky: square; MB-38: circle; Arsenio: triangle; L27PS3: cross); the symbol colours represent the bitter-seed parent accession (Gr56: red; La646: green; La246: blue; LAP123: magenta).

An analysis of population structure was performed by a discriminant principal components analysis (DPCA). In its first step, the DPCA was applied to identify the optimal number of genotype groups (K) for imputation in the GWAS and GS analyses by the k-means algorithm applied to increasing levels of K, selecting the value of K = 17 that minimized the Bayesian Information Content (BIC) (Figure 2, top panel). The final DPCA performed according to this K value retained seven components according to the a-score criterion. The main results of this analysis are summarised in Figure 2 (bottom panel) as a function of the genotype scores in the space of the first two DPCA components. As expected, all genotypes were grouped according to the cross they derived from. The genotypes issued from the four crosses involving Lucky as the sweet-seed parent (square symbols in Figure 2), and those issued from the four crosses having the landrace La646 from the Canary Islands (green symbols in Figure 2), were clearly separated from the rest of the genotypes, whereas the material from crosses of the remaining parents created well-defined but occasionally intertwined clusters (Figure 2).

**Figure 3:**
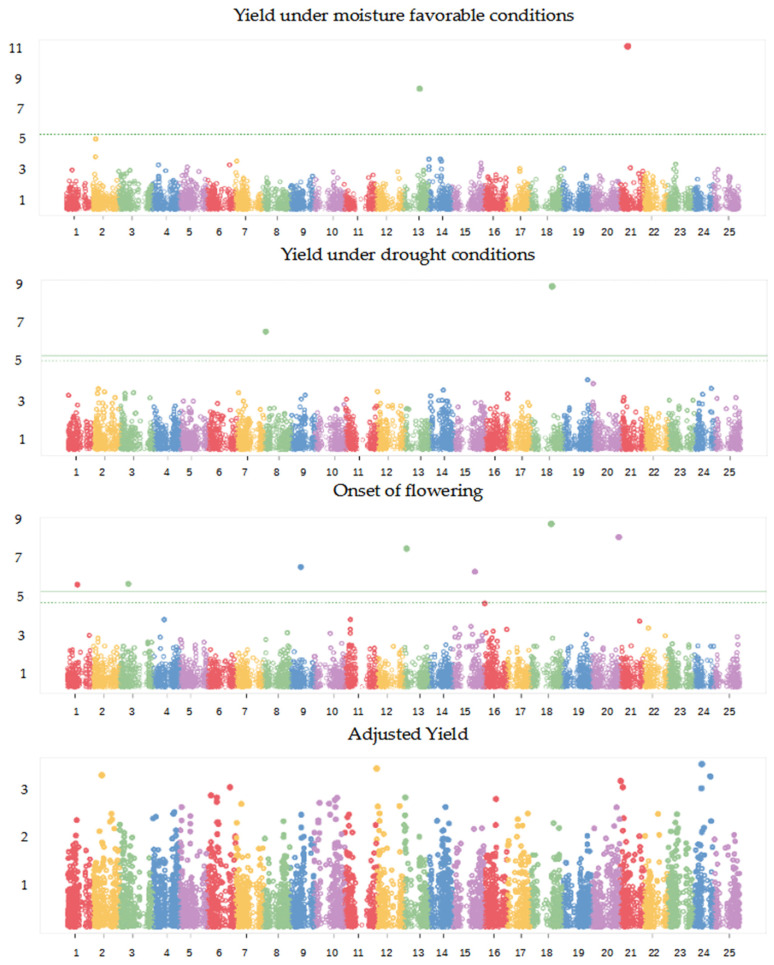
Manhattan plots showing the association scores between 9828 SNPs and grain yield under moisture-favourable and drought stress conditions, onset of flowering (Flowering), and the adjusted grain yield (as deviation from yield under stress expected from linear regression as a function of onset of flowering), for 134 white lupin inbred lines. The dashed and continuous lines represent Bonferroni’s threshold at *p* < 0.05 and *p* < 0.01, respectively.

The GWAS revealed two significant SNPs for grain yield under moisture-favourable conditions on chromosomes 13 and 21, two SNPs for grain yield under severe drought stress on chromosomes 8 and 18, and seven SNPs for the onset of flowering placed in as many different chromosomes (1, 3, 9, 13, 15, 18, and 20) (Figure 3; Table 5). No significant association was detected for the adjusted grain yield (Figure 3). Our results suggested a definite polygenic control not only for the adjusted grain yield but also for yield in either cropping condition, when considering that the proportion of the total phenotypic variance explained by the significant SNPs amounted to 36.8% and 40.5% for grain yield under favourable and stress conditions, respectively (Table 5). The seven significant SNPs for the onset of flowering accounted jointly for over 50% of the trait phenotypic variation (Table 5). Importantly, the most significant SNP marker for grain yield under drought stress, which mapped on chromosome 18, coincided with the most significant SNP for the onset of flowering (Table 5), confirming at the genomic level the association of early phenology with better crop performance under severe drought that emerged from the analysis of phenotypic data.

### 2.3. Genome-Enabled Predictions

We assessed the predictive ability of GS models as Pearson’s correlations between true and predicted phenotypes by envisaging a single-environment predicting scenario for the four traits (via standard cross-validation) as well as a cross-environment predicting scenario for grain yield (where one environment was used to train the model for trait prediction in the other environment). Table 6 reports for each trait the best combination of four tested statistical models, three thresholds of maximum missing rate per SNP marker (0.15, 0.20, 0.30), and the presence or absence of inputted population structure that allowed to maximize the predictive ability. In general, we found a high single-environment predictive ability for all traits, with values ranging from 0.619 for the adjusted grain yield to 0.780 for grain yield in favourable conditions. Predictive ability values displayed relatively modest variation among most combinations of statistical models, missing rate and presence or absence of population structure (Supplementary Figure S2). The cross-environment predictive ability for grain yield was lower than the single-environment scenario but still moderately high in absolute terms, ranging from 0.506 (GS training on moisture-favourable data) to 0.600 (GS training on drought stress data) (Table 6).

The weighted G-BLUP (WGBLUP) statistical model was top-performing for three traits out of six in Table 6, whereas Bayesian Reproducing Kernel Hilbert Space (RKHS) and Bayesian Lasso were top performing for two traits and one trait, respectively. However, the four tested statistical models performed quite similarly in a more thorough model comparison based on predictive ability values averaged across traits (Table 7). Likewise, no threshold of missing rate per SNP marker (0.15, 0.20 or 0.30) provided a consistent predictive ability advantage on the ground of selected top-performing models (Table 6), or predictive ability values averaged across traits (Table 7). Inputting population structure into prediction models did not provide a consistent advantage according to top-performing models, which included this information layer in three cases out of six (Table 6).

## 3. Discussion

The grain yield reduction of 61% arising from the imposed drought stress was severe but largely comparable, for example, with yield reductions in the range of 35–76% that were reported for narrow-leafed lupin across moisture-favourable and drought-prone environments of Australia [34]. The observed extent of GEI for grain yield across moisture-contrasting environments was in substantial agreement with earlier studies on white lupin that were performed in managed environments on germplasm sets mainly composed of accessions [17] or individual genotypes [18] of landrace germplasm. These earlier studies revealed genetic correlation values for yield responses across managed environments that were somewhat lower than the current one (*r_g_* = 0.76–0.77 vs. 0.84). The imposition of greater yield reduction under stress (nearly 80%) and the wider genetic variation of the tested germplasm in those studies may account for this minor difference. A reason for the moderately high genetic correlation across drought-prone and moisture-favourable environments that was observed in this study and the earlier ones could be a general impact of climatic adaptation, since the delay in fulfilling the vernalization requirement of later-flowering lines under the adopted late-winter sowing could disfavour a priori these lines in both managed environments [17]. The occurrence of such a delay is supported by the fact that mean daily temperatures averaged 7.6 °C in the first two weeks following the sowing, while 6 °C would be needed for the vernalization of mid- to late-flowering genotypes in the same period [35]. 

Correlation and regression results for the onset of flowering confirmed that drought stress escape by early flowering is an important mechanism for white lupin adaptation to environments with severe drought. This result was confirmed at the genomic level by the fact that the most important SNP for grain yield under stress coincided with that for the onset of flowering. The association of drought tolerance with early phenology emerged in earlier studies on white lupin [17] and other grain legumes, such as narrow-leafed lupin [36] and pea [22]. The study on pea, which was performed on three sets of recombinant inbred lines under managed stress conditions similar to the current ones, revealed a nearly identical response pattern of line grain yield as a function of the onset of flowering when averaging its results across the three line sets, namely, an average yield loss of 0.033 t/ha per day of delayed flowering (compared with the current value of 0.036 t/ha) across a 20-day range of line onset of flowering that is identical to the current one. The only difference between the two studies was the greater *R*^2^ for the regression of yield as a function of the onset of flowering in the pea study (which averaged 0.63, compared with the current value of 0.29), suggesting that drought stress escape had about two-fold greater impact on grain yield variation of pea lines compared with current lupin lines. The adjusted yield, which was indicative of intrinsic drought tolerance, had lower relative importance in the pea study (where it related to 37% of the total grain yield variation based on the average *R^2^* value) than in the current study (where it related to 71% of the total variation), but its reported broad-sense heritability was quite similar in the two studies (0.57 vs. 0.59). Importantly, the exploitation of the adjusted yield through marker-assisted selection or GS produced pea genotypes with distinctly improved drought tolerance in a following proof-of-concept study [27]. Actually, stress escape by earlier flowering emerged as well as a plant plasticity mechanism in the present study, based on the modest anticipation of mean flowering date exhibited unanimously by all lupin genotypes (without GEI).

Although appealing for drought tolerance improvement, the exploitation of early onset of flowering is limited in many white lupin cropping environments, such as the autumn-sown ones in inland areas of the Iberian, Italian and Balkan peninsulas, because the early-flowering germplasm is more subjected than the late one to winter low-temperature stress and winter mortality [21]. The ideal plant type in these environments ought to combine moderate lateness of flowering with high intrinsic drought tolerance. Indeed, the landrace accession La646, which possesses these characteristics [17], exhibited an exceptionally wide adaptation pattern that made it the top-yielding one out of 121 landraces or modern cultivars evaluated across drought-prone or cold-prone autumn-sown environments of southern Europe [16]. The progeny lines of this landrace clearly tended to inherit these characteristics (Table 4), reinforcing the high value of this accession as a genetic resource for breeding programs. The heritability of intrinsic drought tolerance is also supported indirectly by the mean response of the progeny lines of the Greek landrace Gr56, an accession that exhibited high susceptibility to drought [17]. The indications of heritable variation from parent genotypes to progeny lines and moderately high broad-sense heritability that emerged for complex traits, such as intrinsic drought tolerance and grain yield under severe drought, are encouraging for drought tolerance improvement of this crop.

Intrinsic drought tolerance may rely on different physiological mechanisms in white lupin, such as stomatal closure, greater stomatal conductance, or less reduced net photosynthesis under stress [13,37]. In addition, the ability to accumulate assimilates in the shoots upon drought stress intensification may contribute to plant survival and seed filling under stress through re-translocation [13,38]. This latter mechanism may have contributed to the greater increase of the harvest index under stress that represented a key feature of drought-tolerant lines in this study and emerged already for landrace germplasm [17]. 

We observed a faster LD decay in our germplasm set compared with that reported earlier for a collection of cultivars and landraces [39], possibly because of the generation by our crossing scheme of a high number of heterozygous loci differently combined depending on the specific geographically-contrasting parental lines. The occurrence of substantial variation among chromosomes for LD decay was already found in [39]. Although challenged by the fast LD decay and the somewhat suboptimal genotype sample size, our study was able to reveal a few QTLs for grain yield under moisture-favourable or drought stress conditions while confirming the expected polygenic control of the crop yield traits, especially the adjusted yield (to which various physiological mechanisms with fairly limited individual impact may be expected to contribute). The polygenic control of all yield traits was confirmed by (a) the ability of the significant SNPs to account for only a minor portion of the phenotypic variation and (b) the definitely lower proportion of phenotypic variation that these SNPs could explain compared with that of GS models (which can also account for minor gene effects). An insufficient GWAS power to detect small-effect SNPs [40] likely hindered our ability to identify QTLs with modest genetic effects for grain yield in drought or favourable conditions and the adjusted grain yield.

Interestingly, the significant SNPs for grain yield differed across managed environments in spite of the moderately high genetic correlation across environments. This result emphasized the partly different genetic control of the yield trait in the two cropping conditions. On the other hand, the moderately high genetic correlation for genotype yield response across the contrasting environments, and the only moderate decrease of GS predictive ability for grain yield passing from single-environment to cross-environment prediction scenarios (Table 6), suggested that many small-effect loci for grain yield may be coincident between favourable and drought stress environments.

Our GWAS results suggested a polygenic control also for the onset of flowering, a result that agrees with recent unpublished results by Rychel et al. (personal communication). Some of the significant SNPs revealed by our study mapped on the same chromosomes of QTLs reported in previous GWAS and linkage mapping studies conducted under various vernalization conditions, including regions on chromosomes 1 [41], 3 [42], 13 [26,41,42], and 20 [42]. In contrast with earlier studies [26,41,42], we found no QTL on chromosomes 2 and 16 for this trait, possibly because extremely late-flowering lines were absent from our germplasm set. 

Our study revealed several genomic regions of potential interest for grain yield and onset of flowering selection by scanning a region as long as the mean chromosome distance at which LD dropped below 0.2 in both directions from each significant SNP (Supplementary Table S1). Inter alia, Lalb_Chr13g0291541 that was associated with SNP Chr13_1653492 for the onset of flowering encodes a transcription factor of the C2H2 family [43], which is known to play a role in flowering regulation [44]. However, the polygenic control of all focus traits does support the exploitation of SNP information mainly by means of GS models, also in view of the high predictive ability exhibited by these models. In particular, our findings reinforced the high potential interest of GS for lupin grain yield improvement by confirming for a genetically-broad reference population of sweet-seed breeding lines the high predictive ability of GS found in landrace germplasm [18]. That study reported predictive ability values for single-environment grain yield predictions in moisture-favourable or drought stress conditions in the range of 0.47–0.58, which are somewhat lower than the current range of 0.67–0.78 (Table 7). Likewise, the cross-environment predictive ability values across moisture-favourable and drought-stress environments in [18] (in the range of 0.42–0.51) are somewhat lower than the current values (in the range of 0.51–0.60; Table 6). If the limited seed market size of white lupin supported the breeding of this crop for wide adaptation [45], the moderate consistency of GS predictions across the moisture-contrasting environments could be exploited to build a comprehensive GS model trained on grain yield data from the contrasting environments. On the other hand, the selection for specific adaptation to drought-prone cropping environments could exploit (a) the GS model for grain yield under drought when targeting a mild-winter region or (b) the GS model for the adjusted yield (also featuring a substantial predictive ability), when targeting autumn-sown, relatively cold-prone environments by selection for intrinsic drought tolerance without affecting the phenology.

In conclusion, this study generated information that could support the phenotypic and genomic selection of white lupin for drought-prone or climatically-diversified target regions. It confirmed the importance of an early phenology for drought stress escape on the ground of phenotypic and GWAS results. However, it revealed the presence of heritable, polygenic genetic variation for intrinsic drought tolerance that could be exploited through phenotypic selection or, less expensively, through moderately reliable genomic predictions. It also indicated the feasibility of phenotypic or genomic selection for wide adaptation to moisture-contrasting target environments. The high predictive ability of the current GS models for drought tolerance will be verified by future proof-of-concept work aiming to assess actual genetic gains obtained from GS application to independent germplasm sets.

## 4. Materials and Methods

### 4.1. Plant Material

The plant material for this study included 138 sweet-seed inbred lines chosen from a reference population developed by CREA to broaden the genetic base for white lupin breeding in Europe. The list of test lines and information on their parent germplasm are provided in Supplementary Table S2. The reference population originated from crosses of each of four elite, sweet-seed cultivars or breeding lines with each of four elite, bitter-seed landrace accessions. To further broaden the population genetic base, we used a different parent genotype within a landrace accession for each of its crosses with sweet-seed genotypes (assuming that landraces are genetically heterogeneous, unlike modern cultivars). Landrace accessions were selected out of a world germplasm collection evaluated for grain yield under spring-sowing in France and autumn-sowing in two climatically-contrasting Italian sites [16]. Additional information for parent choice was provided from other studies, e.g., [46] for lime tolerance, [45] for genotype adaptation across Italian environments, and [17] for drought tolerance. In brief, the landrace accessions identified as La246 and La646 in INRAE’s white lupin germplasm collection originated respectively from Italy and the Canary Islands, the French variety Lucky, and the Italian variety Arsenio (referred to as line 7–50 in earlier studies), were selected because of their wide adaptation to climatically-contrasting and/or moisture-contrasting environments; the Moroccan breeding line L27PS3, because of its good adaptation to drought stress environments; the Greek landrace accession Gr56 from INRAE’s collection and the breeding line MB-38, because of the high tolerance to low winter temperatures; and the Italian landrace LAP123 collected by CREA, because of the moderate lime tolerance. Seed quality characteristics contributed to parent choice, e.g., the high γ-conglutin content of Arsenio and Lucky or the very large seed of LAP123. Crosses in 2014 and on-season or off-season single-seed descent-based generations of F_2_ to F_5_ inbred lines for each of the 16 crosses from 2015 to 2017 were carried out in isolation under insect-proof nets to prevent any out-crossing. Within-cross selection for low alkaloid content was performed (a) on F_3_ and F_4_ individual seeds by the fluorescence method [47]; and (b) on F_4_ individual seeds by a non-destructive test that adapted to single seeds the spectrophotometer method described by [48] and [49], in order to discard material whose alkaloid content belonged to the highest 25% quartile. The final population included 960 F_5_ inbred lines (60 per cross), of which 560 (35 per cross) were genotyped, and 192 (12 per cross) were multiplied in isolation in 2018 to obtain F_6_ seed used for this study and to characterize phenologically these lines. The final set of 138 test lines was assembled by randomly choosing within early-maturing crosses and by selecting for earliness within late-maturing crosses in order to avoid the presence in the panel of definitely winter-type germplasm (since a late phenological type was expected a priori to be poorly adapted to severely drought-prone environments). The number of lines per cross ranged from 3 to 10 (Supplementary Table S2). However, the number of lines issued by each individual parent was more balanced, ranging from 27 for MB-38 to 37 for La246, La646, Lucky, Arsenio, and L27PS3.

### 4.2. Phenotyping

The 138 inbred lines were grown in Lodi, northern Italy, in a phenotyping platform already used for other drought-tolerance studies (e.g., [27]). The platform is composed of eight independent, large (24.0 m × 1.6 m × 0.8 m deep) bottomless containers in concrete filled with local soil, covered by a rainout shelter and equipped with a double-rail irrigation boom (Supplementary Figure S3). Four containers formed just as many complete replicates of a managed environment with imposed severe drought stress; the other four represented the replicates of a moisture-favourable managed environment. The containers were filled with local sub-acid (pH 6.5), sandy-loam (55.9% sand, 32.4% silt, 11.7% clay) soil, which featured 22.2% (in volume) field capacity and 9.0% (in volume) wilting point. Each plot included two rows of five plants, each spaced 0.15 m across and within rows (plant density = 44 plants/m^2^), keeping two edge plants as border plants. Sowing took place in the late winter (mid-February) in 2019 to avoid any confounding effect of susceptibility to low temperatures in addition to drought. Mineral fertilization was incorporated into the seedbed at the rates of 27 kg/ha of N, 46 kg/ha of P_2_O_5_, and 50 kg/ha of K_2_O. The seed was inoculated with NPPL HiStick inoculant (Becker Underwood, Toulouse, France) prior to sowing. Pre-emergence weed control was performed by applying 1.5 L/ha of Stomp Aqua (Basf Agro; Pendimentalin 38%). After an initial stage of favourable vegetative growth that implied 120 mm of irrigation, two conditions of water availability were imposed, starting from 16 April to 16 June (i.e., one to two weeks before crop harvest). A Diviner 2000 capacitance sensor (Sentek Pty Ltd., Stepney, Australia) monitored the soil moisture every two to three days. In the moisture-favourable environment, irrigation of about 20 mm was applied when the soil water content decreased beneath 60% of the maximum available soil water (i.e., soil water content at field capacity minus soil water content at the wilting point) in the upper 40 cm, bringing back the water content to a level exceeding 80% of the maximum available soil water. In the drought-stress environment, an irrigation of about 10 mm was applied at the soil water content corresponding to the wilting point, replenishing 15% of the maximum available soil water. The moisture-favourable environment received a total of 240 mm of irrigation in 12 applications, whereas the stress environment was irrigated five times with a total of 50 mm. Daily mean temperatures and daily maximum temperatures averaged 12.4 °C and 17.3 °C, respectively, in April, 14.3 °C and 19.3 °C in May, and 24.0 °C and 29.8 °C in June.

We recorded on a plot basis the onset of flowering as the number of days from 1 April to when 50% of the plants displayed three open flowers. Dry grain yield, dry straw biomass, and harvest index (as the ratio of grain to aerial biomass) were assessed after harvesting and threshing the whole plot at crop maturity and oven drying the grain and the straw at 60 °C for four days to constant weight. 

### 4.3. Statistical Analysis of Phenotyping Data

An ANOVA including the fixed factor genotype and the random factor block was performed for each trait in each environment to assess the significance of the variation among lines and its extent as the genetic coefficient of variation computed as:

CV = (*s_g_*/*m*) × 100 (1)

where, *m* is the trait mean value, and *s_g_* is the square root of the genotypic component of variance (*s*^2^*_g_*) estimated along with the experimental error (*s*^2^*_e_*) component of variance by a restricted maximum likelihood (REML) method. Trait broad-sense heritability was computed from these components of variance by the equation:

*H*^2^ = *s*^2^*_g_*/(*s^2^_g_* + *s*^2^*_e_*/*n*) (2)

where, *n* is the number of experiment replicates, computing an approximate standard error as reported in [50]. We used *H*^2^ values to compute the best linear unbiased prediction (BLUP) values according to [51], which were used for GS analyses.

A second ANOVA including the fixed factor environment and the random factors genotype and block within environment was performed for each trait to test the variation relative to water treatments, genotypes, and GEI, assessing the extent of the last two effects by estimating the respective components of variance through a REML method. The consistency of the genotype responses across environments was assessed in terms of genetic correlation as described in [52] for one trait assessed in different environments. We tested each genetic correlation coefficient for statistical differences to unity to verify the occurrence of inconsistent response across environments on the ground of confidence intervals computed by multiplying standard errors according to [53] by relevant *t* values.

We verified whether genotype grain yields under severe drought were affected by the onset of flowering through a regression analysis, assessing the significance of linear and curvilinear responses. Line mean yields of flowering across environments were used in this analysis, given the lack of GEI observed for this trait. In the presence of a significant inverse linear response, we estimated for each line an ‘adjusted’ grain yield on a plot basis as the deviation of its actual yield from the yield value expected for the line as a function of its onset of flowering in the linear regression model, as described in an earlier study on pea [22]. Such adjusted grain yield (which had negative or positive values according to the deviation direction and averaged zero) enabled an ANOVA comparison of the lines for grain yield under stress after removing the mean effect of drought escape as determined by differences in phenology, thereby focusing on grain yield as affected essentially by drought tolerance mechanisms. In addition, we estimated the drought susceptibility index (DSI) proposed by Fischer and Maurer [32]:

DSI = [1 − (*Y_S_*/*Y_F_*)]/*D_S_* (3)

where, *Y_S_* and *Y_F_* stand for line grain yield under stress and favourable conditions, respectively, and *D_S_* is an index of drought severity estimated from irrigation water over the crop cycle for stress (*I_S_*) and favourable (*I_F_*) conditions:

*D_S_* = (*I_F_* − *I_S_*)/*I_F_* (4)

No ANOVA could be applied to DSI because its values were estimated from cultivar mean values within each condition. We assessed the line variation for DSI values in terms of phenotypic CV.

Simple correlation analyses were used to assess (a) interrelationships between traits within each environment and (b) patterns of covariation for GEI effects of different traits. The mean value of inbred line progenies issued from each parent provided an estimate of the parent value for yield under severe drought. All analyses were carried out using the SAS/STAT^®^ software [54].

### 4.4. DNA Isolation, GBS Library Construction, and Sequencing

Genomic DNA was extracted from young leaves of F_5_ plants of each inbred line using the DNeasy Plant Mini Kit (Qiagen, Milan, Italy). Nucleic acid was quantified by a Quant-iT™ PicoGreen™ dsDNA Assay Kit (P7589, Life Technologies, Italy), checking its quality by 1% agarose gel electrophoresis. A trial digestion was carried out on 10% of the DNA samples using the Optizyme EcoRI restriction enzyme (25,000 U, Fisher BioReagents, Rodano, MI, Italy), to compare bands of cut and uncut DNA. The reaction was performed at 37 °C for one hour and the enzyme was deactivated at 65 °C for 20 min. DNA samples were sent to The Elshire Group Ltd. laboratory (Palmerston North, New Zealand) for outsourced library preparation and sequencing. GBS data were generated according to Elshire et al.’s [25] method with the following changes: we used 100 ng of genomic DNA and 3.6 ng of total adapters and restricted the genomic DNA with *Ape*KI enzyme (NEB New England Biolabs, R0643L); then, the library was amplified with Kapa Taq polymerase Alpha (KAPA Library Amplification Readymix, Kapa Biosystems KK2611) by 14 PCR cycles. Sequencing was performed on a single Illumina HiSeq X lane, at 2X150 bp paired-end. Adopting *Ape*KI as the restriction enzyme according to [25] was supported by the fact that about 60% of the white lupin genome includes repetitive DNA sequences [33], which this enzyme tends to skip.

### 4.5. Genotype SNP Calling Procedures, Data Filtering and Imputation

GBS raw reads were demultiplexed using Axe demultiplexer [55]. Trimming for restriction enzyme remnants, alignment on the reference genome and SNP calling were performed using the dDocent pipeline [56]. For alignment, we used the *Lupinus albus* genome version 1.0 [33], which was downloaded from https://www.whitelupin.fr/ (accessed on 3 November 2022). The final genotype matrix, in the form of a vcf file, was further filtered for quality using the vcftools software [57] with parameters −*minQ 30 −max-non-ref-af 1 –non-ref-af 0.001*. The resulting data set was filtered for monomorphic markers, minor allele frequency (MAF) > 5%, missing SNP marker rate < 10%, 20% or 30%, and a missing rate per individual < 50%. Following Nazzicari et al. [58], we estimated missing data by random forest imputation [59] using the R package MissForest [60] with the configuration ntree = 100, maxiter = 10 and encoding genotypes as categorical data (factors).

Both genotypes and SNPs were filtered for excess heterozygosis by using the mean plus three and two standard deviations as maximum thresholds, respectively.

### 4.6. Analysis of Population Structure and Genome-Wide Association Study

The presence and pattern of population structure were investigated by a discriminant principal components analysis (DPCA) [61]. We used the k-means clustering algorithm iteratively for increasing values of K genotype groups from 1 to 20 to identify the optimal number of groups according to the local minimum of the Bayesian information criterion (BIC). The analyses were performed on the output of an ordinary principal components analysis performed on SNP data to benefit from its dimensionality reduction but keeping all the components to avoid information loss. We performed the final DPCA after selecting the optimal K value. The optimal number of DPCA axis to retain for the following analyses was selected by the a-score criterion (which represents the propensity of DPCA toward overfitting). The whole procedure was implemented using the R package *adegenet* [62] using functions *find.clusters(), dapc() and optim.a.score()*.

Linkage disequilibrium (LD) was estimated for each chromosome in R as the squared Pearson’s correlation (*r*^2^) between all pairwise combinations of SNPs within a 50 kb window from genotype data filtered by 0.3 missing data per marker, 0.5 missing data per sample, 0.05 MAF, and excess SNP and genotype heterozygosis, plotted against physical distance, and fitted by a polynomial curve, as described in [63].

A GWAS was performed for grain yield under both water treatments, the onset of flowering averaged across the two water treatments, and the adjusted grain yield using 9828 mapped SNPs according to the Blink model [64] in R package GAPIT3 [65]. The first seven components of a DPCA with K = 17 were included in the GWAS, as they properly account for the population structure based on the visual inspection of quantile-quantile plots comparing the observed trait-marker association scores with those expected in the case of no significant association (Supplementary Figure S4). The statistical significance of trait-marker associations was assessed by Bonferroni’s threshold at *p* < 0.01.

### 4.7. Genome-Enabled Predictions

We tested several whole-genome regression models for the focus traits (grain yield in both favourable and stress conditions, onset of flowering averaged across the two water treatments, and adjusted grain yield) considering four possible statistical models described below, the presence or absence of population structure inputted as in the GWAS, and three thresholds of maximum missing rate per SNP marker (0.15, 0.20 and 0.30). We first envisaged a single-environment (alias intra-environment) prediction scenario, assessing the predictive ability of GS models by standard 10-fold cross-validation. Each model was tested twice, repeating the analyses ten times and reporting the average results to ensure numerical stability. We also envisaged a cross-environment prediction scenario for grain yield assessed in the favourable and stress environments, using by turns one environment for training and the other for validation (by splitting the training data in a 90/10 fashion as done for single-environment predictions). The whole procedure was repeated ten times for numerical stability.

We considered four possible whole-genome regression models: Ridge Regression BLUP (rrBLUP), Bayesian Lasso (BL), Bayesian Reproducing Kernel Hilbert Space (RKHS), and Weighted G-BLUP (WGBLUP). All models were implemented using R package GROAN [66].

The rrBLUP model [67] assumes a linear mixed additive model where each marker is assigned an effect as a solution of the following equation:

y = 1μ + Wq + ε(5)

where, y is the vector of observed phenotypes; μ is the mean of y; W is the genotype matrix (e.g., {0,1,2} for biallelic SNPs); q ~ N (0, Iσ^2^_q_) is the vector of marker effects; and **ε** is the vector of residuals. The model is solved in a maximum likelihood context estimating the ridge parameter λ = σ^2^_e_/σ^2^_q_ representing the ratio between residual and markers variance. When the population structure is included in the model the above formula is updated as follows:

y = 1μ + Wq +Xb + ε(6)

where, X is the is structure matrix with one row per sample and one column per considered DPCA component and b ~ N (0, Iσ^2^_b_) is the vector of (fixed) effects corresponding to the population structure.

BL [68] solves the same general model than rrBLUP but in the bayesian context where regression parameters have independent Laplace double-exponential priors. The system is solved via Gibbs sampling with proper iteration count (10,000 repetitions) and burn-in period (1000 repetitions) so as to ensure convergence. When present, the population structure was added to the model as a fixed (i.e., flat prior) component.

RKHS model is used to solve the so-called genomic BLUP (G-BLUP) in the Bayesian context. First, a genomic kinship additive matrix G is computed following [69]. The matrix is then used in the following model:

y = 1μ + Zg + ε(7)

where, Z is a design matrix allocating samples to genetic values and g is a vector of additive genetic effects for a sample with var(g) = Gσ^2^_g_ where G is the genomic relationship matrix and σ^2^_g_ is the genetic variance for this model. In the context of RKHS the G matrix is considered as the reproducing kernel function mapping from each pair of markers to covariance. The system is then solved with a standard Gibbs sampling as done in BL, with the same configuration to accommodate for population structure if required.

WGBLUP [70] is very similar to RKHS and operatively follows the same implementation, with the main difference being that matrix G is substituted by matrix G*, which is computed weighing the SNP markers by the *p* values resulting from an association study. The association scores were computed programmatically inside each cross-validation cycle on the training set using statgenGWAS R package [71]. Once the scores were obtained, the G* matrix was computed as:

G* = ZDZ′/[2Σp*_i_* (1 − p*_i_*)] (8)

where, Z is an identity matrix for the markers; D is a diagonal matrix where each element of the diagonal corresponds to SNP weights; and p*_i_* is the observed MAF of all genotyped individuals.

## Figures and Tables

**Table 1:** Mean value, genetic or phenotypic coefficient of variation (CV), and broad-sense heritability on a line mean basis (*H*^2^), for 138 white lupin inbred lines in drought stress and moisture-favourable managed environments.

Trait	Environment ^a^	Mean ^b^	CV (%) ^c^	*H* ^2^
Grain yield (t/ha)	Stress	0.88	28.7	0.70 ± 0.04
Grain yield (t/ha)	Favourable	2.28	35.1	0.69 ± 0.04
Straw biomass (t/ha)	Stress	2.25	19.6	0.65 ± 0.05
Straw biomass (t/ha)	Favourable	6.85	30.4	0.72 ± 0.04
Aerial biomass (t/ha)	Stress	3.13	20.4	0.65 ± 0.05
Aerial biomass (t/ha)	Favourable	9.13	30.6	0.71 ± 0.04
Harvest index	Stress	0.281	10.9	0.76 ± 0.03
Harvest index	Favourable	0.250	10.2	0.58 ± 0.06
Onset of flowering (days from 1 April)	Stress	38.7	11.2	0.93 ± 0.01
Onset of flowering (days from 1 April)	Favourable	41.5	10.9	0.92 ± 0.01
Adjusted grain yield ^d^ (t/ha)	Stress	0	−	0.59 ± 0.06
Drought susceptibility index (DSI) ^e^	−	1.08	29.2	−

^a^ Water treatments applied from 15 April to 15 June. Favourable: soil water content in the range of 60–80% of max. available content; stress: soil water content ranging from wilting point to 15% of max. available content. ^b^ Trait means difference between environments significant at *p* < 0.10 for harvest index and onset of flowering and at *p* < 0.01 for the other traits. ^c^ CV was not estimable for adjusted grain yield (due to zero trait value), was phenotypic for DSI (computed on within-environment line mean values), and was genetic for the other traits. Variation among lines significant at *p* < 0.01 for all traits, including the adjusted grain yield. ^d^ As deviation from yield under stress expected from linear regression as a function of onset of flowering; used as indicator of intrinsic drought tolerance. ^e^ According to [32].

**Table 2:** Genotype (G) and genotype × environment interaction (GEI) components of variance, and genetic correlation (*r_g_*) of genotype values across environments, for traits of 138 white lupin inbred lines grown in drought stress and moisture-favourable managed environments.

	Variance Component ^a^		
Trait	G	GEI	*r_g_* ± SE
Grain yield (t/ha)	0.170	0.182	0.84 ± 0.07
Straw biomass (t/ha)	0.857	1.414	0.93 ± 0.07
Aerial biomass (t/ha)	1.675	2.448	0.93 ± 0.07
Harvest index	0.000557	0.000242	0.71 ± 0.09
Onset of flowering (days from 1 April)	19.98	0	1.00 ± 0.01

^a^ All components different from zero at *p* < 0.01 except GEI for onset of flowering.

**Table 3:** Trait phenotypic correlations for 138 white lupin inbred lines grown in drought stress and moisture-favourable managed environments.

Trait	Grain Yield, Stress	Adjusted Grain Yield, Stress	Grain Yield, Favourable
Straw biomass ^a^	0.71 **	0.69 **	0.86 **
Aerial biomass ^a^	0.87 **	0.79 **	0.92 **
Harvest index ^a^	0.73 **	0.49 **	0.59 **
Onset of flowering ^a^	−0.54 **	0.00	−0.40 **
Drought susceptibility index (DSI) ^b^	−0.15 †	−0.14 †	0.62 **
Adjusted grain yield ^c^	0.84 **	−	−

†, ** = different from zero at *p* < 0.10 and *p* < 0.01, respectively. ^a^ Relative to the growing condition in which grain yield was assessed. ^b^ According to [32]. ^c^ As deviation from yield under stress expected from linear regression as a function of onset of flowering; used as indicator of intrinsic drought tolerance.

**Table 4:** Breeding value of eight white lupin parent genotypes used for 16 factorial crosses of landrace × sweet-seed cultivar or breeding line germplasm based on mean values of their progeny lines for grain yield under severe drought and adjusted yield, and mean progeny values of onset of flowering.

Plant Material	Parent Genotype	Yield (t/ha)	Adjusted Yield (t/ha) ^a^	Onset of Flowering (dd from 1 April)
Landrace	Gr56	0.731	−0.170	38.1
Landrace	LAP123	0.984	0.035	36.8
Landrace	La246	0.885	0.006	38.8
Landrace	La646	0.944	0.105	39.9
Cultivar/Breeding line	Arsenio	0.892	0.024	39.1
Cultivar/Breeding line	L27PS3	0.942	−0.016	36.6
Cultivar/Breeding line	Lucky	0.941	0.051	38.4
Cultivar/Breeding line	MB-38	0.769	−0.084	39.5

^a^ As deviation from yield under stress expected from linear regression as a function of onset of flowering.

**Table 5:** Significant (*p* < 0.01) SNPs detected by a GWAS based on 9828 SNPs and 134 white lupin inbred lines for grain yield under moisture-favourable (Yield_NS) and drought conditions (Yield_S), and for onset of flowering (Flowering), with proportion of explained phenotypic variance and minor allele frequency (MAF).

SNP	Trait	Variance (%)	MAF
Chr21_5357552	Yield_NS	29.0	0.10
Chr13_10513725	Yield_NS	7.8	0.43
Chr18_13052982	Yield_S	31.9	0.12
Chr08_862028	Yield_S	8.6	0.24
Chr18_13052982	Flowering	20.3	0.12
Chr20_17362727	Flowering	1.3	0.42
Chr13_1653492	Flowering	2.1	0.22
Chr09_6606393	Flowering	9.2	0.11
Chr15_14142372	Flowering	1.1	0.49
Chr03_5647810	Flowering	10.4	0.05
Chr01_6340005	Flowering	5.8	0.06

**Table 6:** Predictive ability (as Pearson’s correlation between true and predicted phenotypes) of best-performing genomic selection models for single-environment (first four rows) and cross-environment (last two rows) prediction scenarios.

Trait ^a^	Model ^b^	Population Structure Included	Maximum Missing Rate per SNP Marker	Predictive Ability ^c^
Onset of flowering	WGBLUP	Yes	0.30	0.760
Grain yield [favourable]	WGBLUP	Yes	0.30	0.780
Grain yield [stress]	WGBLUP	Yes	0.20	0.670
Adjusted grain yield	RKHS	No	0.15	0.619
Grain yield [favourable] → Grain yield [stress]	BL	No	0.15	0.506
Grain yield [stress] → Grain yield [favourable]	RKHS	No	0.15	0.600

^a^ In the cross-environment scenario, the first reported trait (before the arrow) is used for training the model to predict the second trait. ^b^ WGBLUP, Weighted G-BLUP; RKHS, Bayesian Reproducing Kernel Hilbert Space; BL, Bayesian Lasso. ^c^ The training procedure was repeated 10 times, reporting the mean values.

**Table 7:** Comparison of four statistical models for genomic selection based on their predictive ability averaged across the six traits (four according to a single-environment scenario and two according to a cross-environment scenario) listed in Table 6, for different thresholds of allowed missing rate per SNP marker.

Model ^a^	Maximum Missing Rate per SNP Marker			
	0.15	0.20	0.30	Average
BL	0.630	0.628	0.628	0.629
RKHS	0.640	0.638	0.644	0.641
rrBLUP	0.634	0.635	0.636	0.635
WGBLUP	0.636	0.634	0.638	0.636
Average	0.635	0.634	0.636	0.635

^a^ BL, Bayesian Lasso; RKHS, Bayesian Reproducing Kernel Hilbert Space; Ridge Regression BLUP; WGBLUP, Weighted G-BLUP.

## Data Availability

Data available in Data repository 1 of Supplementary Materials.

## Supplementary Materials

Supplementary Materials can be downloaded at: https://www.mdpi.com/article/10.3390/ijms24032351/s1. Table S1: List of genes potentially associated with the significant SNPs detected by a GWAS based on 9828 SNPs and 134 white lupin inbred lines for grain yield under moisture-favourable (Yield_NS) and drought conditions (Yield_S), and onset of flowering (Flowering), identified by scanning a region as long as the mean chromosome distance at which LD dropped to 0.2 in both directions from each significant SNP and reported with the relative annotated function (https://www.whitelupin.fr/; accessed on 3 November 2022). Table S2: List of 138 white lupin test inbred lines and their parent germplasm. Figure S1: LD decay plots for white lupin chromosomes based on Pearson’s correlation (*r^2^*) (Y-axis) and physical distance (X-axis) estimated on pairwise combinations of 9828 SNPs within a 50 kb window for 134 inbred lines. Figure S2: Comparison of genome-enabled predictions. Each panel represents regressions on one of the studied traits, either in a single-environment scenario (top four panels) or a cross-environment scenario (bottom two panels). In the cross-environment scenario, the first reported trait (before the arrow) is used for training the model to predict the second trait. On the x-axis, the maximum allowed level of missing rate per SNP marker is reported. On the y-axis, the predicted ability, as the Pearson’s correlation between true and predicted phenotypes, is reported. Line colours represent different regression models. Dashed-line models include information on population structure as derived from DPCA; continuous-line models do not include population structure. Figure S3: Portion of the phenotyping platform including four large, bottomless containers, in which moisture-favourable and drought-stress water treatments were assigned to outer and inner containers, respectively, from 16 April until 16 June. Figure S4: Quantile-quantile plots comparing the observed trait-marker association scores with those expected in case of no significant association for a GWAS based on 9828 SNPs and 134 white lupin inbred lines and performed for grain yield under moisture-favourable (Yield_NS) and drought conditions (Yield_S), onset of flowering (Flowering), and adjusted grain yield (Adjusted Yield). Data repository S1: Genotypic data (file S1_SNP_markers.csv), phenotypic data (file S2_phenotypes.csv), square matrix of kinships computed using the Astle and Balding method (file S3_kinship.csv), and first seven DPCA components resulting after clustering with K = 17 (file S4_DPCA.csv), and legends of data repository files.

## Abbreviations

ANOVA	Analysis of variance
BL	Bayesian Lasso
RKHS	Bayesian Reproducing Kernel Hilbert Space
DPCA	Discriminant principal components analysis
DSI	Drought susceptibility index
GEI	Genotype × environment interaction
GBS	Genotyping-by-sequencing
GS	Genomic selection
QTL	Quantitative trait locus
REML	Restricted maximum likelihood
rrBLUP	Ridge Regression BLUP (best linear unbiased prediction)
SNP	Single nucleotide polymorphism
WGBLUP	Weighted G-BLUP
