# Supplementary material for: White Lupin Drought Tolerance: Genetic Variation, Trait Genetic Architecture, and Genome-Enabled Prediction
Source: Int J Mol Sci. 2023 Jan 25;24(3):2351. doi: 10.3390/ijms24032351 (PMC9916572; doi:10.3390/ijms24032351)
Supplement: Supplementary file 1 [file ijms-24-02351-s001.zip › ijms-2142144-supplementary/supplementary Figure S1.pdf]

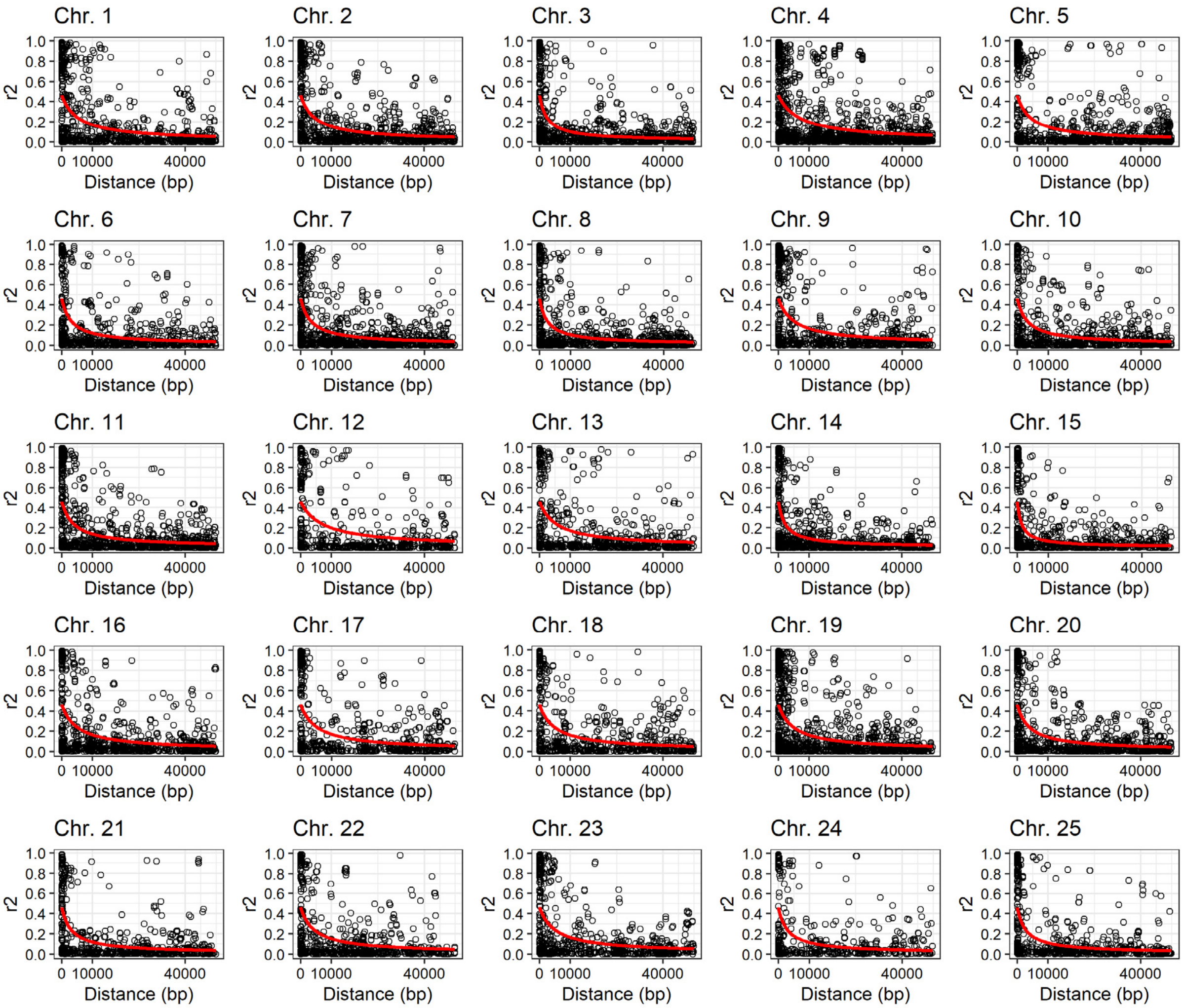

Supplementary Figure S1. LD decay plots for white lupin chromosomes based on Pearson's correlation (r^2) (Y axis) and physical distance (X axis) estimated on pairwise combinations of 9,828 SNPs within a 50 kb window for 134 recombinant inbred lines.
