# Supplementary material for: White Lupin Drought Tolerance: Genetic Variation, Trait Genetic Architecture, and Genome-Enabled Prediction
Source: Int J Mol Sci. 2023 Jan 25;24(3):2351. doi: 10.3390/ijms24032351 (PMC9916572; doi:10.3390/ijms24032351)
Supplement: Supplementary file 1 [file ijms-24-02351-s001.zip › ijms-2142144-supplementary/supplementary Figure S2.pdf]

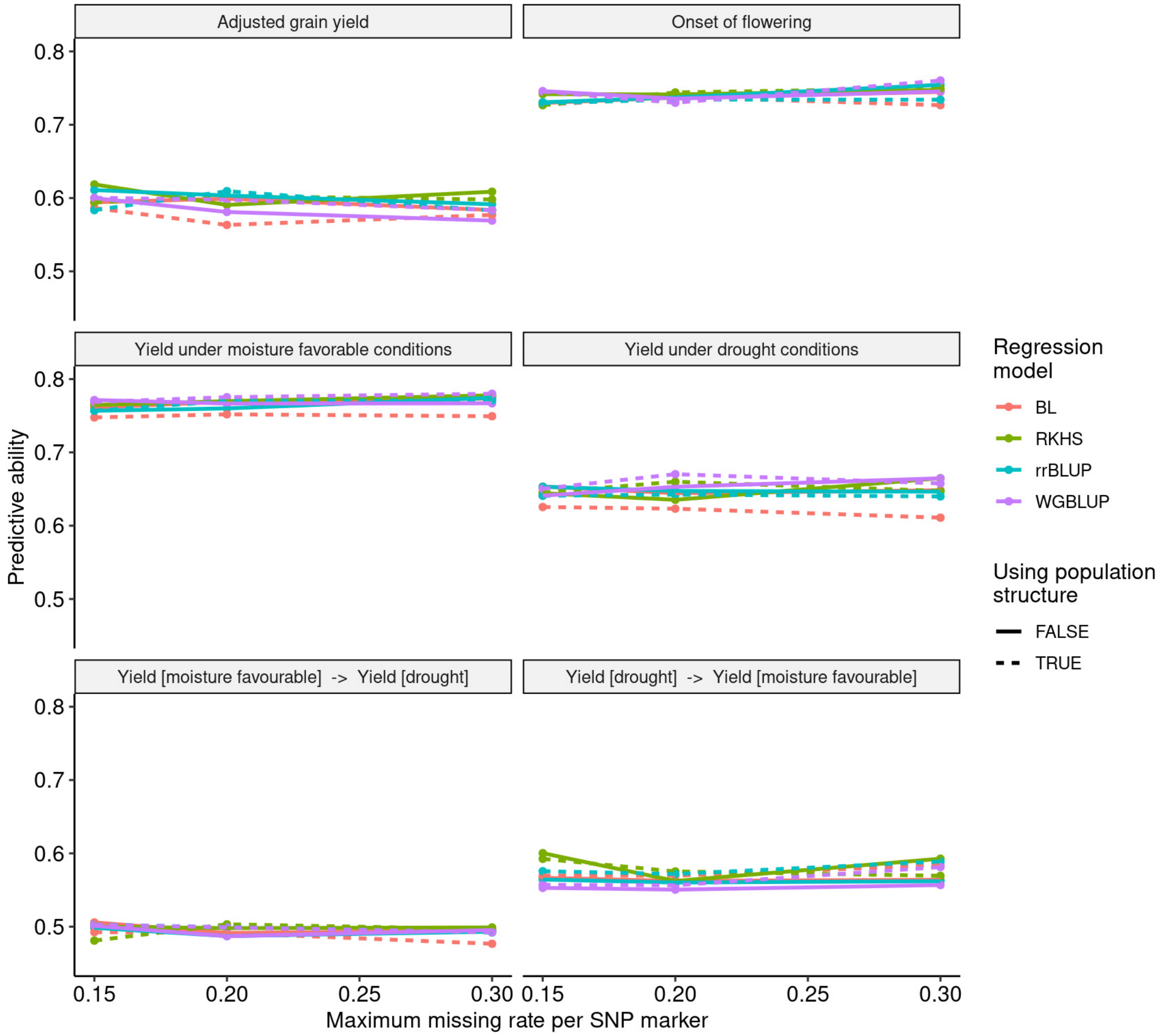

Supplementary Figure S2. Comparison of genome-enabled predictions. Each panel represents regressions on one of the focus traits, either in a *single-environment* scenario (top four panels) or *cross-environment* scenario (bottom two panels). In the *cross-environment* scenario, the first reported trait (before the arrow) is used for training the model to predict the second trait. The x-axis reports the maximum allowed level of missing rate per SNP marker; the y-axis reports the predicted ability as Pearson's correlation between true and predicted phenotypes. Line colours represent different regression models. Dashed-line models include information on population structure as derived from DPCA; continuous-line models do not include population structure. BL, Bayesian Lasso; RKHS, Bayesian Reproducing Kernel Hilbert Space; Ridge Regression BLUP; WGBLUP, Weighted G-BLUP.
