# Supplementary material for: White Lupin Drought Tolerance: Genetic Variation, Trait Genetic Architecture, and Genome-Enabled Prediction
Source: Int J Mol Sci. 2023 Jan 25;24(3):2351. doi: 10.3390/ijms24032351 (PMC9916572; doi:10.3390/ijms24032351)
Supplement: Supplementary file 1 [file ijms-24-02351-s001.zip › ijms-2142144-supplementary/supplementary Figure S3.pdf]

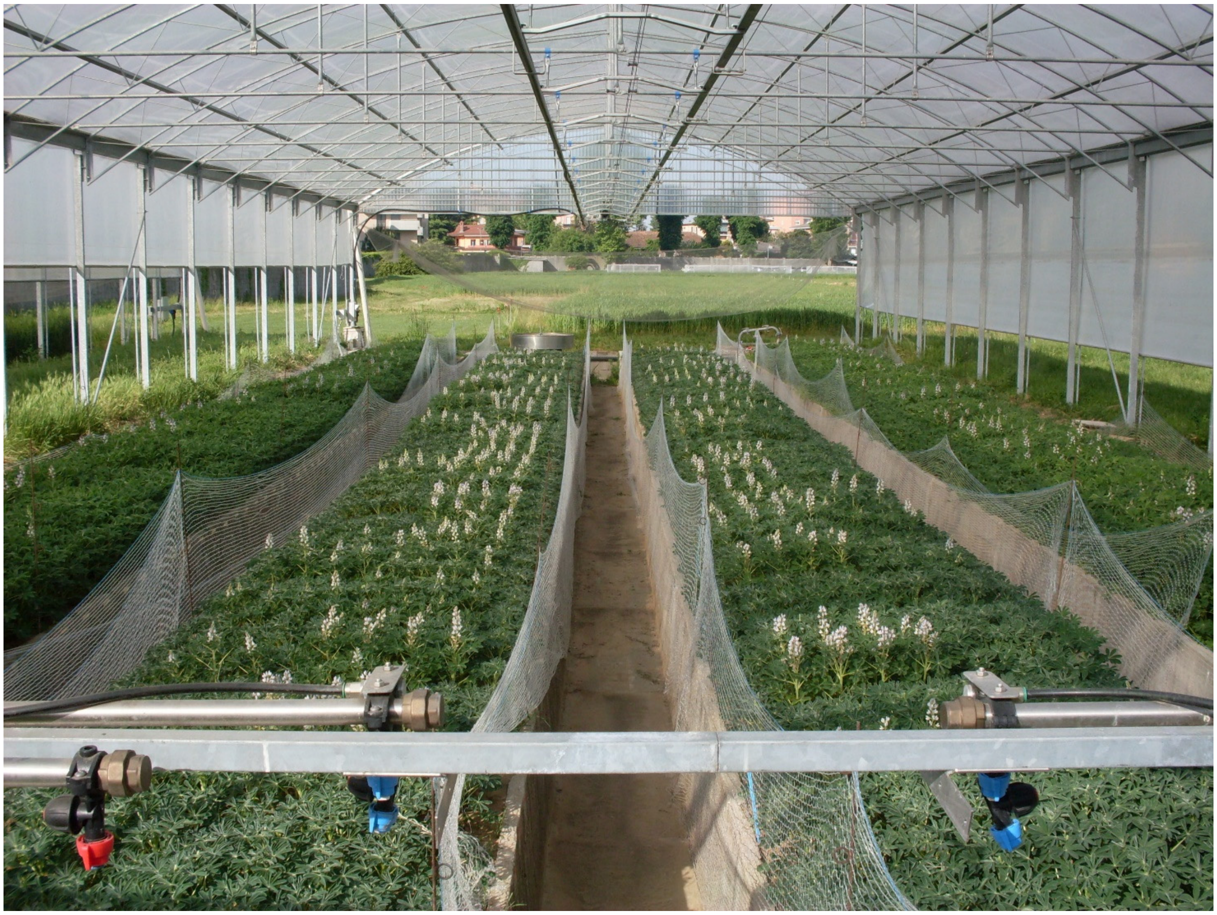

Supplementary Figure S3. Portion of the phenotyping platform including four large, bottomless containers, in which moisture-favourable and drought stress water treatments were assigned to outer and inner containers, respectively, from April 16 until June 16.
