# Supplementary material for: White Lupin Drought Tolerance: Genetic Variation, Trait Genetic Architecture, and Genome-Enabled Prediction
Source: Int J Mol Sci. 2023 Jan 25;24(3):2351. doi: 10.3390/ijms24032351 (PMC9916572; doi:10.3390/ijms24032351)
Supplement: Supplementary file 1 [file ijms-24-02351-s001.zip › ijms-2142144-supplementary/supplementary Figure S4.pdf]

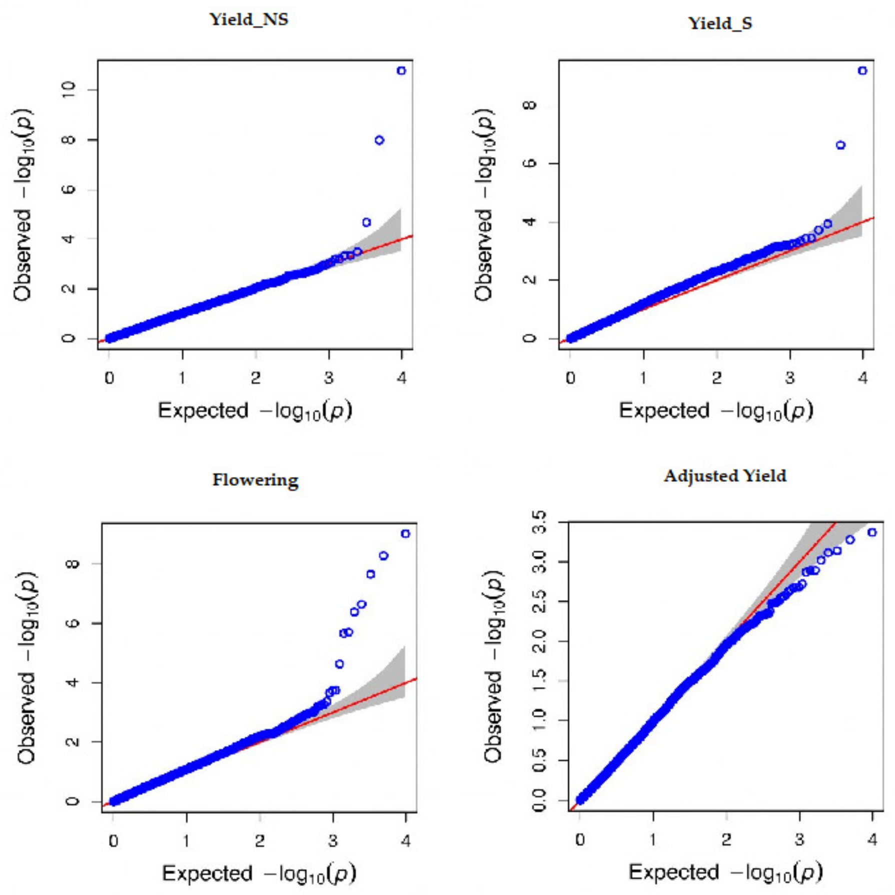

Supplementary Figure S4. Quantile-quantile plots comparing the observed trait-marker association scores with those expected in case of no significant association, for a GWAS based on 9,828 SNPs and 134 white lupin recombinant inbred lines performed for grain yield under moisture-favorable (Yield_NS) and drought conditions (Yield_S), onset of flowering (Flowering), and adjusted grain yield as yield deviation from the expected value according to the linear regression of yield as a function of onset of flowering.
