# Supplementary material for: White Lupin Drought Tolerance: Genetic Variation, Trait Genetic Architecture, and Genome-Enabled Prediction
Source: Int J Mol Sci. 2023 Jan 25;24(3):2351. doi: 10.3390/ijms24032351 (PMC9916572; doi:10.3390/ijms24032351)
Supplement: Supplementary file 1 [file ijms-24-02351-s001.zip › ijms-2142144-supplementary/supplementary Table S1.pdf]

Supplementary Table S1. List of genes potentially associated to the significant SNPs detected by a GWAS based on 9,828 SNPs and 134 white lupin inbred lines for grain yield under moisture favorable (Yield_NS) and drought conditions (Yield_S), and onset of flowering (Flowering), identified by scanning a region as long as the mean chromosome distance at which LD dropped to 0.2 in both directions from each significant SNP and reported with the relative annotated function (https://www.whitelupin.fr/).

SNP	Scanned Interval	Trait	Gene	Function
Chr01_6340005	Chr01_6332535:6347475	Flowering	Lalb_Chr01g0009841	Putative inositol-polyphosphate 5-phosphatase
Chr01_6340005	Chr01_6332535:6347475	Flowering	Lalb_Chr01g0009851	Putative small GTPase superfamily, P-loop containing nucleoside triphosphate hydrolase
Chr03_5647810	Chr03_5644341:5651279	Flowering	Lalb_Chr03g0034071	Putative multi antimicrobial extrusion protein
Chr08_862028	Chr08_858391:865665	Yield_S	Lalb_Chr08g0231661	Putative clathrin, heavy chain
Chr09_6606393	Chr09_6599110:6613676	Flowering	Lalb_Chr09g0330521	Putative peroxin-3
Chr09_6606393	Chr09_6599110:6613676	Flowering	Lalb_Chr09g0330531	Putative pre-mRNA-splicing factor SPF27
Chr13_10513725	Chr13_10506098:10521352	Yield_NS	Lalb_Chr13g0295531	Putative transcription factor Hap2/NF-YA family
Chr13_10513725	Chr13_10506098:10521352	Yield_NS	Lalb_Chr13g0295541	Putative small monomeric GTPase
Chr13_10513725	Chr13_10506098:10521352	Yield_NS	Lalb_Chr13g0295551	Putative YTH domain-containing protein
Chr13_1653492	Chr13_1645865:1661119	Flowering	Lalb_Chr13g0291541	Putative transcription factor C2H2 family

Chr13_1653492	Chr13_1645865:1661119	Flowering	Lalb_Chr13g0291551	Putative small-subunit processome, Utp11 protein
Chr15_14142372	Chr15_14140358:14144386	Flowering	Lalb_Chr15g0082751	Putative oxidoreductase
Chr18_13052982	Chr18_13046123:13059841	Yield_S; Flowering	Lalb_Chr18g0052061	hypothetical protein
Chr18_13052982	Chr18_13046123:13059841	Yield_S; Flowering	Lalb_Chr18g0052071	hypothetical protein
Chr20_17362727	Chr20_17357046:17368408	Flowering	Lalb_Chr20g0122361	Putative cadmium-transporting ATPase
Chr20_17362727	Chr20_17357046:17368408	Flowering	Lalb_Chr20g0122371	Putative transcription factor interactor and regulator C3H-WRC/GRF family
Chr21_5357552	Chr21_5353306:5361798	Yield_NS	Lalb_Chr21g0313091	hypothetical protein
Chr21_5357552	Chr21_5353306:5361798	Yield_NS	Lalb_Chr21g0313101	Putative mitogen-activated protein MAP kinase phosphatase
