# Supplementary material for: White Lupin Drought Tolerance: Genetic Variation, Trait Genetic Architecture, and Genome-Enabled Prediction
Source: Int J Mol Sci. 2023 Jan 25;24(3):2351. doi: 10.3390/ijms24032351 (PMC9916572; doi:10.3390/ijms24032351)
Supplement: Supplementary file 1 [file ijms-24-02351-s001.zip › ijms-2142144-supplementary/supplementary Table S2.pdf]

Supplementary Table S2. List of 138 white lupin test inbred lines and their parent germplasm.

Line	Cross	Female parent	Male parent		Line	Cross	Female parent	Male parent
1.09	1	Lucky	Gr56		9.09	9	Lucky	La246
1.22	1	Lucky	Gr56		9.16	9	Lucky	La246
1.31	1	Lucky	Gr56		9.42	9	Lucky	La246
1.44	1	Lucky	Gr56		9.54	9	Lucky	La246
1.51	1	Lucky	Gr56		9.58	9	Lucky	La246
1.62	1	Lucky	Gr56		9.60	9	Lucky	La246
1.71	1	Lucky	Gr56		9.78	9	Lucky	La246
1.74	1	Lucky	Gr56		9.81	9	Lucky	La246
1.87	1	Lucky	Gr56		9.86	9	Lucky	La246
2.56	2	MB-38	Gr56		10.03	10	MB-38	La246
2.70	2	MB-38	Gr56		10.18	10	MB-38	La246
2.91	2	MB-38	Gr56		10.21	10	MB-38	La246
3.03	3	Arsenio	Gr56		10.39	10	MB-38	La246
3.07	3	Arsenio	Gr56		10.40	10	MB-38	La246
3.09	3	Arsenio	Gr56		10.44	10	MB-38	La246
3.17	3	Arsenio	Gr56		10.47	10	MB-38	La246
3.35	3	Arsenio	Gr56		10.59	10	MB-38	La246
3.38	3	Arsenio	Gr56		10.62	10	MB-38	La246
3.39	3	Arsenio	Gr56		10.91	10	MB-38	La246
3.42	3	Arsenio	Gr56		11.01	11	Arsenio	La246
3.75	3	Arsenio	Gr56		11.02	11	Arsenio	La246
4.05	4	L27PS3	Gr56		11.09	11	Arsenio	La246
4.07	4	L27PS3	Gr56		11.10	11	Arsenio	La246
4.12	4	L27PS3	Gr56		11.22	11	Arsenio	La246
4.23	4	L27PS3	Gr56		11.23	11	Arsenio	La246
4.25	4	L27PS3	Gr56		11.25	11	Arsenio	La246
4.53	4	L27PS3	Gr56		11.37	11	Arsenio	La246
4.56	4	L27PS3	Gr56		11.40	11	Arsenio	La246
4.66	4	L27PS3	Gr56		12.09	12	L27PS3	LAP123
4.67	4	L27PS3	Gr56		12.12	12	L27PS3	LAP123
5.06	5	Lucky	La646		12.15	12	L27PS3	LAP123
5.12	5	Lucky	La646		12.25	12	L27PS3	LAP123
5.23	5	Lucky	La646		12.34	12	L27PS3	LAP123
5.28	5	Lucky	La646		12.36	12	L27PS3	LAP123
5.34	5	Lucky	La646		12.37	12	L27PS3	LAP123
5.40	5	Lucky	La646		12.44	12	L27PS3	LAP123
5.43	5	Lucky	La646		12.50	12	L27PS3	LAP123
5.50	5	Lucky	La646		13.08	13	Lucky	LAP123
5.51	5	Lucky	La646		13.19	13	Lucky	LAP123

6.01	6	MB-38	La646		13.24	13	Lucky	LAP123
6.30	6	MB-38	La646		13.27	13	Lucky	LAP123
6.41	6	MB-38	La646		13.31	13	Lucky	LAP123
6.43	6	MB-38	La646		13.32	13	Lucky	LAP123
6.45	6	MB-38	La646		13.37	13	Lucky	LAP123
6.47	6	MB-38	La646		13.45	13	Lucky	LAP123
6.58	6	MB-38	La646		13.50	13	Lucky	LAP123
6.62	6	MB-38	La646		13.62	13	Lucky	LAP123
6.65	6	MB-38	La646		14.01	14	MB-38	LAP123
6.71	6	MB-38	La646		14.02	14	MB-38	LAP123
7.10	7	Arsenio	La646		14.03	14	MB-38	LAP123
7.25	7	Arsenio	La646		14.04	14	MB-38	LAP123
7.26	7	Arsenio	La646		15.04	15	Arsenio	LAP123
7.31	7	Arsenio	La646		15.05	15	Arsenio	LAP123
7.46	7	Arsenio	La646		15.10	15	Arsenio	LAP123
7.47	7	Arsenio	La646		15.17	15	Arsenio	LAP123
7.54	7	Arsenio	La646		15.29	15	Arsenio	LAP123
7.56	7	Arsenio	La646		15.32	15	Arsenio	LAP123
7.72	7	Arsenio	La646		15.33	15	Arsenio	LAP123
8.02	8	L27PS3	La646		15.36	15	Arsenio	LAP123
8.08	8	L27PS3	La646		15.62	15	Arsenio	LAP123
8.14	8	L27PS3	La646		15.91	15	Arsenio	LAP123
8.18	8	L27PS3	La646		16.10	16	L27PS3	LAP123
8.34	8	L27PS3	La646		16.20	16	L27PS3	LAP123
8.37	8	L27PS3	La646		16.22	16	L27PS3	LAP123
8.49	8	L27PS3	La646		16.29	16	L27PS3	LAP123
8.51	8	L27PS3	La646		16.35	16	L27PS3	LAP123
8.55	8	L27PS3	La646		16.37	16	L27PS3	LAP123
					16.39	16	L27PS3	LAP123
					16.52	16	L27PS3	LAP123
					16.77	16	L27PS3	LAP123
					16.88	16	L27PS3	LAP123
