# Supplementary material for: White Lupin Drought Tolerance: Genetic Variation, Trait Genetic Architecture, and Genome-Enabled Prediction
Source: Int J Mol Sci. 2023 Jan 25;24(3):2351. doi: 10.3390/ijms24032351 (PMC9916572; doi:10.3390/ijms24032351)
Supplement: Supplementary file 1 [file ijms-24-02351-s001.zip › ijms-2142144-supplementary/Data repository S1.zip!/legends of data repository files.pdf]

Legends for supplementary material

- Table S1 (file S1_SNP_markers.csv): matrix of genotypes, one row per sample, one column per SNP marker. Column names indicate the position on the genome. The first column indicates the sample. Values in 0/1/2.
- Table S2 (file S2_phenotypes.csv): matrix of phenotypes. One row per sample, one column per measured trait, either in BLUE or BLUP form. The first column indicates the sample. One extra column reports the cross to which the sample belongs.
- Table S3 (file S3_kinship.csv): square matrix of kinships computed using Astle & Balding method. One row/column per sample.
- Table S4 (file S4_DPCA.csv): the first seven components resulting from DPCA after clustering with K=17
